# Effect of the FA2H Gene on cashmere fineness of Jiangnan cashmere goats based on transcriptome sequencing

**DOI:** 10.1186/s12864-022-08763-7

**Published:** 2022-07-21

**Authors:** Cuiling Wu, Jianying Li, Xinming Xu, Qi Xu, Chongkai Qin, Guifen Liu, Chen Wei, Guoping Zhang, Kechuan Tian, Xuefeng Fu

**Affiliations:** 1 Institute of Animal Science and Veterinary Medicine, Shandong Academy of Agricultural Sciences, Jinan, 250100 China; 2 College of Animal Science, Xinjiang Agricultural University, Urumqi, 830052 China; 3 Key Laboratory of Genetics Breeding and Reproduction of Xinjiang Wool Sheep and Cashmere-Goat, Institute of Animal Science, Xinjiang Academy of Animal Sciences, Urumqi, 830011 China; 4 Key Laboratory of Special Environmental Medicine, Xinjiang Military General Hospital, Urumqi, 830000 China; 5 Xinjiang Aksu Prefecture Animal Husbandry Technology Extension Center, Aksu, 843000 China

**Keywords:** Jiangnan cashmere goats, Cashmere fineness, *FA2H*, Dermal papilla cells

## Abstract

**Background:**

Cashmere goats are a heterogeneous hairy mammal. The fineness of cashmere can affect its economic value. Therefore, in this study, we used transcriptome sequencing techniques to analyze the gene expression profiles of the skin tissues of cashmere goats with different cashmere fineness. The selected candidate genes were functionally verified with the secondary hair follicle hair papillary cells of cashmere goats.

**Results:**

We identified 479 DEGs, of which 238 mRNAs were up-regulated in the fine velvet group and 241 mRNA were down-regulated. Based on functional annotation and protein interaction network analysis, we found some genes that may affect the fineness of cashmere, including *SOX18*, *SOX4*, *WNT5A*, *IGFBP4*, *KAP8*, *KRT36,* and *FA2H*. Using qRT-PCR, Western blot, CCK-8 cell viability detection, EDU cell proliferation detection, and flow cytometry, we found that overexpression of the *FA2H* gene could promote the proliferation of secondary hair follicle DPCs in cashmere goats. At the same time, we proved that *FA2H* could regulate the expression levels of the *FGF5* and *BMP2* genes in DPCs.

**Conclusion:**

The results of this study provide a useful reference for the genetics and breeding of Jiangnan cashmere goats and goat genome annotation, and provide an experimental basis for improving cashmere quality of the cashmere goat.

**Supplementary Information:**

The online version contains supplementary material available at 10.1186/s12864-022-08763-7.

## Background

Cashmere is a high-grade textile raw material that is known to be delicate, smooth, and have a good luster. Cashmere fineness is a quantitative trait that is regulated by micro-effect polygenes. Early studies have shown that the diameter of cashmere fibers is inversely proportional to the visible height of cashmere scales, and is proportional to the density of cashmere scales. With the increase in cashmere fiber diameter, the proportion of positive cortical cells also increases [1]. To understand fiber diameter traits, transcriptomic studies have been carried out on the main cashmere goat breeds in China, such as the Liaoning cashmere goat [2], Inner Mongolia cashmere goat [3, 4], and Tibetan cashmere goat [5, 6]. In genome research on cashmere fiber diameter, most scholars have adopted genome-wide association analysis [7, 8] and candidate gene polymorphism analysis [9, 10] among other strategies. A new breed known as the Jiangnan cashmere goat has had, few reports on its cashmere traits. Therefore, in this study, we performed transcriptome sequencing of the skin tissue of the secondary hair follicles of the Jiangnan cashmere goat, in order to explore the differences in the gene expression profiles of the skin tissue of cashmere goats with coarse cashmere and fine cashmere.

At present, there are few studies on lipid gene regulation of hair follicle growth and development. Fatty Acid 2-Hydroxylase (*FA2H)* is one of the metabolic enzymes of fatty acids. Earlier scholars compared gene expression profiles in different hair follicle cells in mice. Earlier studies found that the *FA2H* gene was expressed in five types of hair follicle cells in mice, including dermal papilla cells (DPCs), dermal fibroblasts, melanocytes, Matrix, and outer root sheath cells. Among them, it is highly expressed in the outer root sheath cells [11]. In addition, studies have shown that *FA2H* deletion causes sebocyte hyperplasia and sebaceous gland enlargement in adult mice. Furthermore, mice lacking *FA2H* exhibited periodic hair loss and alopecia during the telogen phase. These results imply a role for *FA2H* in hair follicle homeostasis [12]. In a study of cashmere goats, *FA2H* was found to be a Hub gene in the network of genes related to hair follicle cycle development by weighted gene co-expression network analysis [13]. This evidence suggested that *FA2H* gene is related to hair follicle development and hair traits. However, the mechanism by which *FA2H* regulates hair follicles remains unclear. Cashmere is produced from the secondary hair follicles of cashmere goats. The DPCs in the secondary hair follicle play a pivotal role as a “signaling center” in hair follicle morphogenesis and cycling [14]. Therefore, verification of the role of FA2H in the DPCs of secondary hair follicles will help to explain the molecular mechanism of FA2H in the regulation of cashmere traits in cashmere goats.

## Results

### Descriptive statistics of fiber diameter traits

Based on the measurement results for the average fiber diameter, eight 24-month-old female Jiangnan cashmere goats were selected as research objects, with four goats in the fine cashmere (Fe) group and four goats in the coarse cashmere (Ce) group. As shown in Table 1, the mean fiber diameter (MFD) of the Fe group was 13.64 ± 0.04 μm, and the MFD of the Ce group was 15.31 ± 0.04 μm. T-test results showed that the MFD values of the Fe group and Ce group were significantly different (*P* < 0.01).

**Table 1:** Summary of individual information and fiber diameter measurement results of experimental goats

Sample Number	Sample Name	Date of birth	Weight/kg	MFD/μm	FDSD	CVFD/%
198,060	Fe-1	2019/3/6	28.50	13.40	3.22	20.35
190,002	Fe-2	2019/3/4	28.80	13.56	3.01	20.89
198,414	Fe-3	2019/2/28	26.50	13.77	3.28	20.39
195,760	Fe-4	2019/2/24	27.40	13.84	3.48	19.95
Mean	Fe	-	27.80 ± 0.53^a^	13.64 ± 0.10^a^	3.25 ± 0.10^a^	20.40 ± 0.19^a^
198,125	Ce-1	2019/2/26	26.80	15.14	3.55	20.13
198,412	Ce-2	2019/2/28	28.50	15.32	3.29	19.92
199,462	Ce-3	2019/3/10	28.00	15.58	3.33	20.34
198,619	Ce-4	2019/3/3	27.50	15.18	3.46	20.11
Mean	Ce	-	27.70 ± 0.36^a^	15.31 ± 0.10^b^	3.41 ± 0.06^a^	20.13 ± 0.09^a^

*Note*: *MFD* stands for mean fiber diameter, *FDSD* represents mean fiber diameter standard deviation, *CVFD* represents coefficient of variation in fiber diameter. Data in the same column with different lowercase letters on the shoulder show significant differences (*P* < 0.05), while the same letters on the shoulder indicate no significant difference (*P* > 0.05)

### Quality control of sequencing data

A total of 92.68 Gb clean data were obtained after RNA sequencing was completed. The clean data of each sample reached 10.88 Gb. The percentage of Q20 of each sample was not less than 98.38%, and the percentage of Q30 was not less than 95.03%. The GC content ranged from 46.92 to 47.49% (Table S1). The quality control results showed that the sequencing results were reliable and suitable for subsequent data analysis. The alignment efficiency of reads of each sample to the reference genome ranged from 96.63 to 97.21% (Table S2).

### Differentially expressed mRNA analysis

A total of 31,251 genes were expressed in 8 Jiangnan cashmere goat skin tissues, of which 10,658 were novel genes. The Fragments Per Kilobase of Transcript per Million Fragments Mapped (FPKM) density distribution of mRNA are shown in Fig. S1. It can be seen from the figure that the FPKM density distribution of eight skin tissue mRNAs was similar, with two peaks, which are concentrated in the order of 10^–1^ and 10^1^ respectively. Boxplots of FPKM distributions of mRNAs are shown in Fig. S2. The gene expression levels of eight skin tissues were relatively concentrated.

**Fig. 1:**
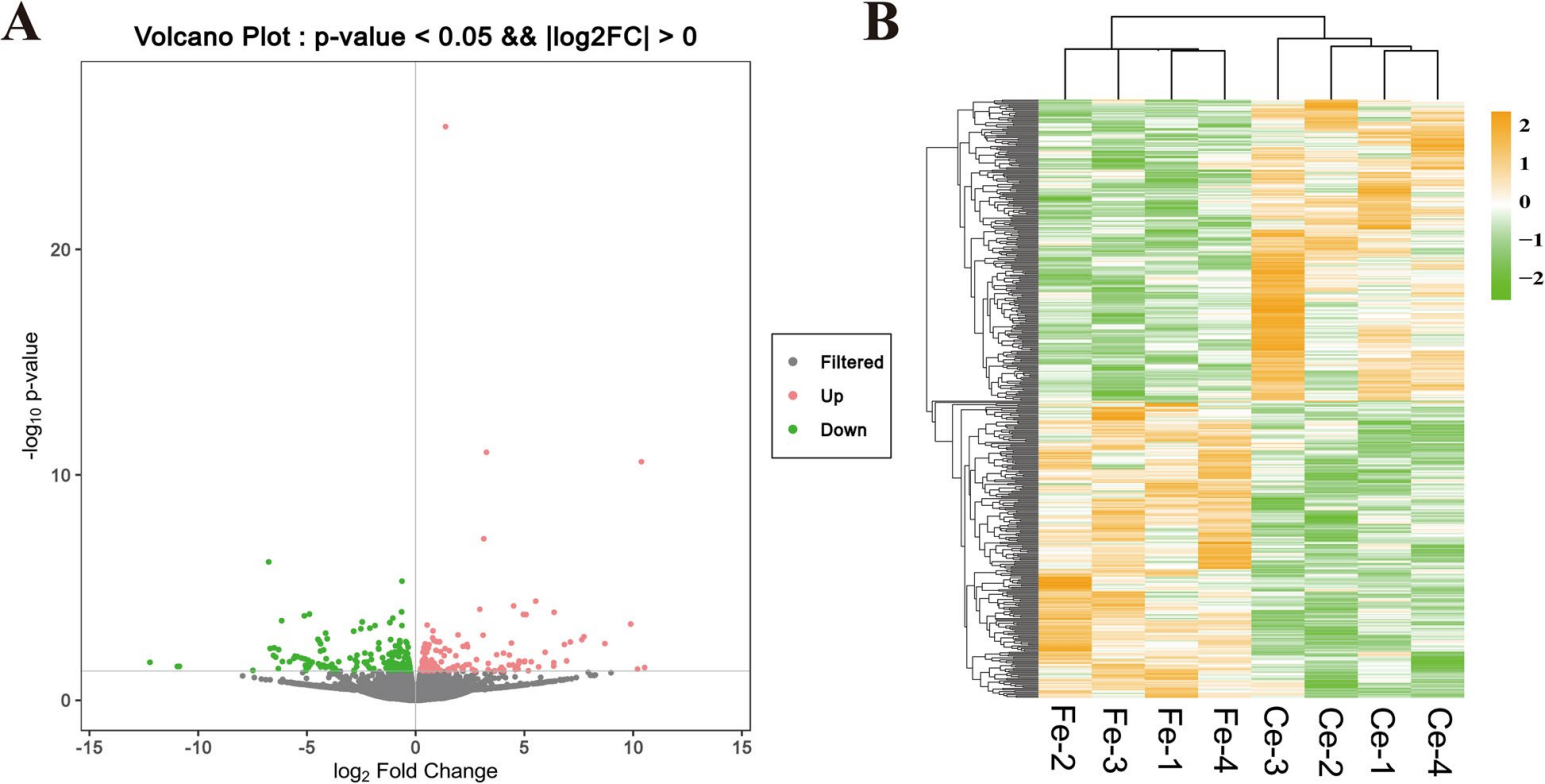
Analysis of DEGs. **A**. Volcano map; **B**. Heatmap

The gene expression profiles of the skin tissues of the Ce group and Fe group were compared. A total of 479 differentially expressed genes (DEGs) were found in the skin tissues of the Ce and Fe groups. Compared with Ce group, 238 mRNAs were up-regulated and 241 mRNAs were down-regulated in Fe group, of which 33 and 15 were novel genes, respectively. (Fig. 1).

### Functional annotation

**Fig. 2:**
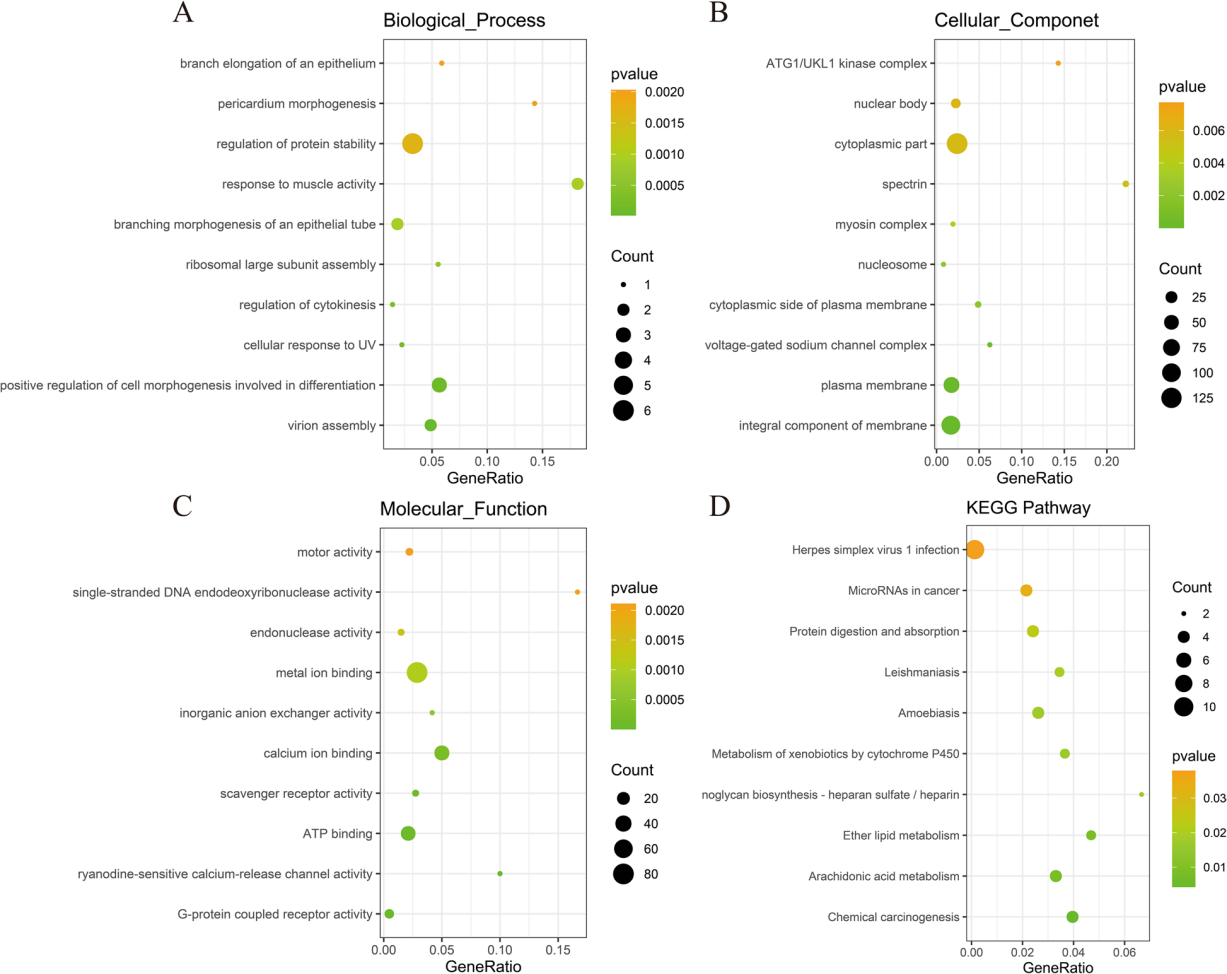
Bubble plots of GO and KEGG enrichment analysis. **A**. Biological process; **B**. Cellular process; **C**. Molecular function; **D**. KEGG pathway analysis

We carried out Gene Ontology (GO) and Kyoto Encyclopedia of Genes and Genomes (KEGG) annotation analysis on the DEGs. The top 10 GO items and pathways with the highest enrichment degree were analyzed (*P* < 0.05). Based on GO analysis, in the classification of biological processes, DEGs was mainly enriched in the virion assembly (GO: 0,019,068), positive regulation of cell morphogenesis involved in differentiation (GO: 0,010,770), and cellular response to UV (GO: 0,034,644) (Fig. 2A). In cellular componet classification, it was mainly enriched in the integral component of membrane (GO: 0,016,021), plasma membrane (GO: 0,005,886), and cytoskeletal part (GO: 0,044,430) (Fig. 2B). Molecular function classification showed it was mainly enriched in the G-protein coupled receptor activity (GO: 0,004,930), ATP binding (GO: 0,005,524), and calcium ion binding (GO: 0,005,509) (Fig. 2C). Similarly, based on KEGG annotation analysis, DEGs was mainly enriched in the Chemical carcinogenesis (ko05204), Arachidonic acid metabolism (ko00590), Ether lipid metabolism (ko00565) (Fig. 2D).

**Fig. 3:**
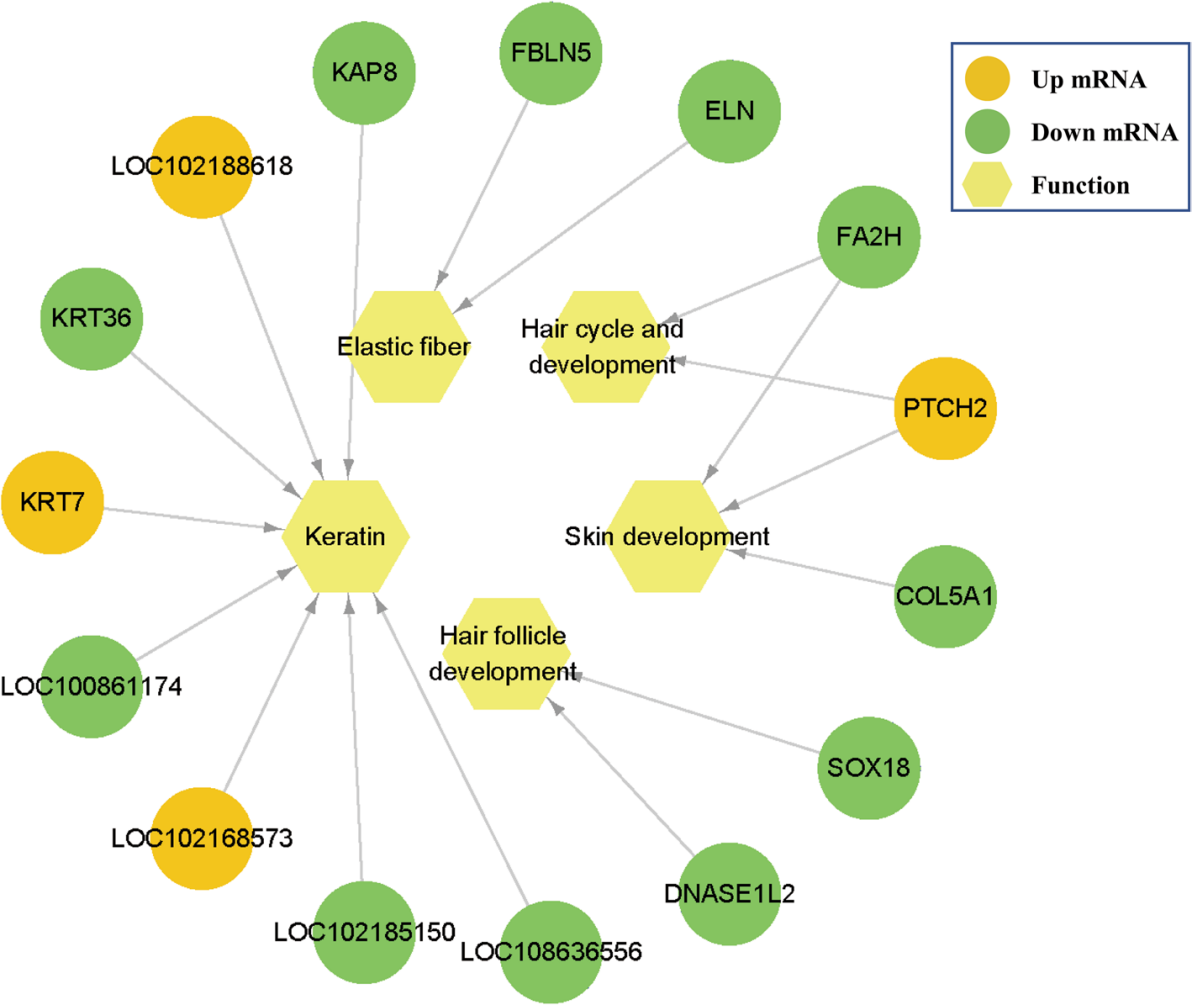
Functional network diagram of DEGs

Combined with the GO analysis results, we focused on some hair and skin-related functional genes, as shown in Fig. 3. *FBLN5* and *ELN* genes were annotated to Elastic fiber (GO:0,071,953). *SOX18* and *DNASE1L2* were annotated to hair follicle development (GO:0,001,942). *FA2H* and *PTCH2* were annotated to regulation of hair cycle (GO:0,042,633, GO:0,042,634). *FA2H*, *PTCH2,* and *COL5A1* were annotated to skin development (GO:0,043,588, GO:0,061,436). In addition, eight keratin family genes were included here, including *LOC102168573*, *LOC108636556*, *LOC102188618*, *LOC100861174*, *LOC102185150*, *KRT36*, *KRT7,* and *KAP8*.

### Protein interaction analysis

**Fig. 4:**
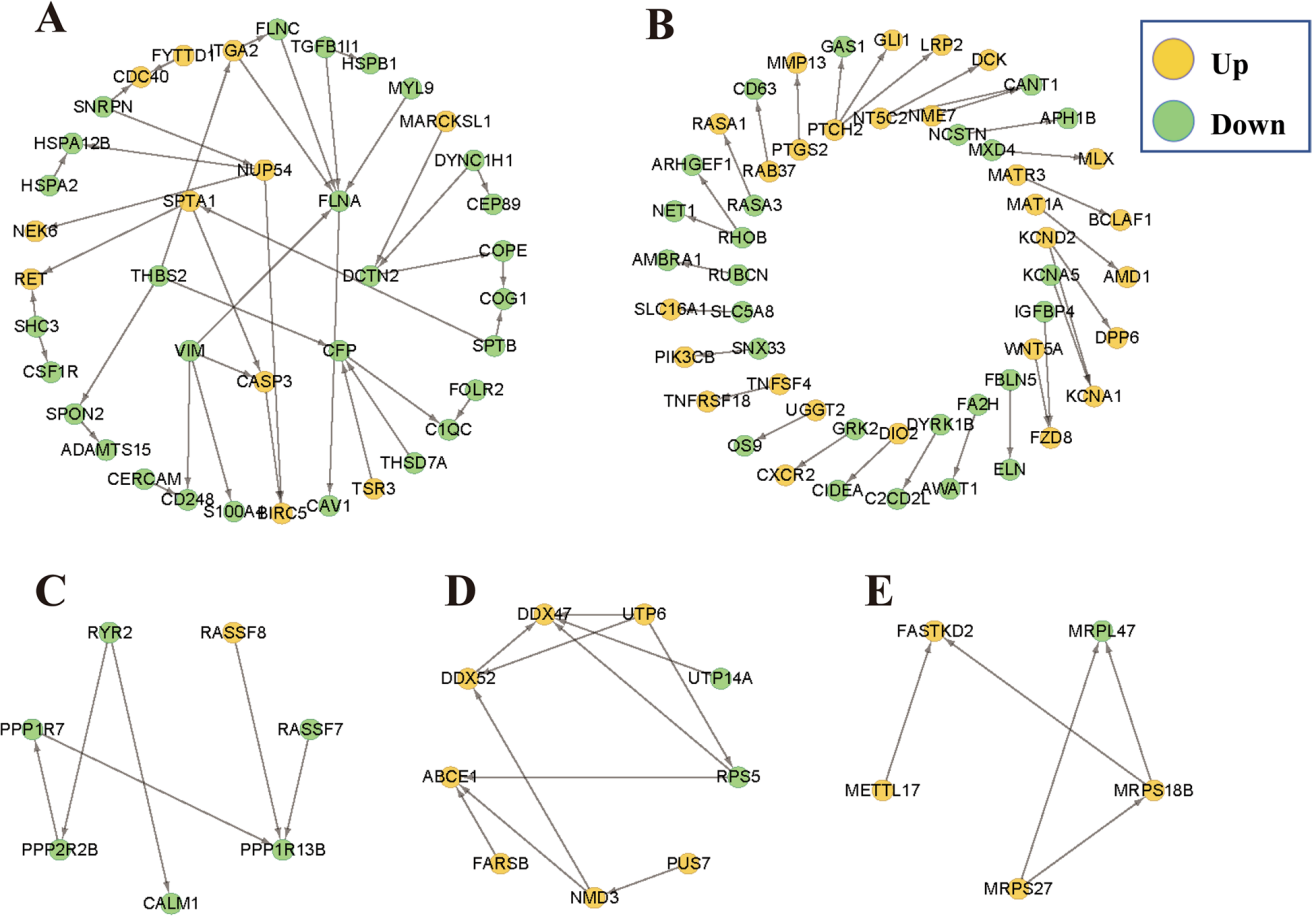
Protein interaction networks of DEGs

We annotated the proteins encoded by known DEGs into the STRING database to construct a protein interaction network. It can be seen from Fig. 4 that when confidence > 0.70, 122 DEGs constructed five protein interaction networks, including 99 pairs of interaction relationships. The average local clustering coefficient was 0.20, and the PPI enrichment p-value was 0.07. Protein network A had the most interactions, in which FLNA was the core node, with a node degree of 6. It is worth noting that FA2H was constructed with protein interaction network B, which is composed of interaction pairs with less than four nodes. Protein network C is mainly composed of Ras association domain family members (RASSF8 and RASSF7) and protein phosphatase family members (PPP1R13B, PPP2R2B, and PPP1R7). The protein network D is composed of DDX47 protein, a member of the DEAD box protein family. The protein network E is mainly composed of the mitochondrial ribosomal protein(MRPS18B).

### *FA2H* expression verification

**Fig. 5:**
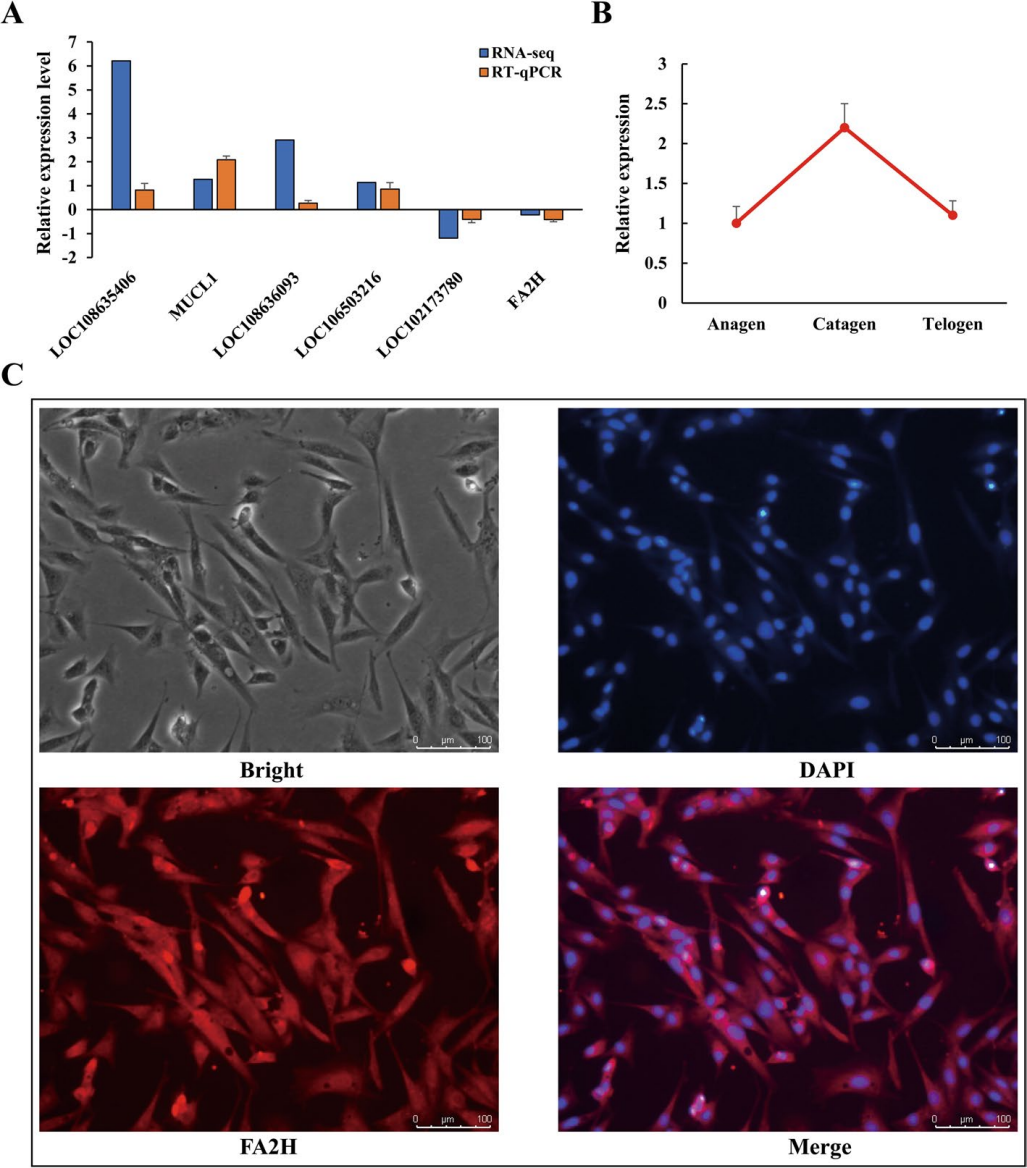
Verification of *FA2H* expression level. **A**. Validation of mRNAs by qRT-PCR; **B**. Expression levels of the *FA2H* gene in the skin tissue of secondary hair follicles at different stages. **C**. FA2H immunofluorescence of DPCs in cashmere goats

To verify the reliability of the RNA-seq data, six DEGs (*LOC108635406*, *MUCL1*, *LOC108636093*, *LOC106503216*, *LOC102173780,* and *FA2H*) were selected for qRT-PCR analysis. It can be seen from Fig. 5A that the qRT-PCR expression level of DEGs is consistent with the trend of RNA-seq data. This indicates that the transcriptome sequencing data are reliable. In addition, the expression level of the *FA2H* gene in the secondary hair follicle catagen phase was higher than that in the anagen phase and telogen phase (Fig. 5B). It can be seen from Fig. 5C that the FA2H protein can be expressed on DPCs using the cell immunofluorescence technique.

### Effects of the *FA2H* gene in DPCs

#### Screening of Interfering Fragments of the *FA2H* Gene

**Fig. 6:**
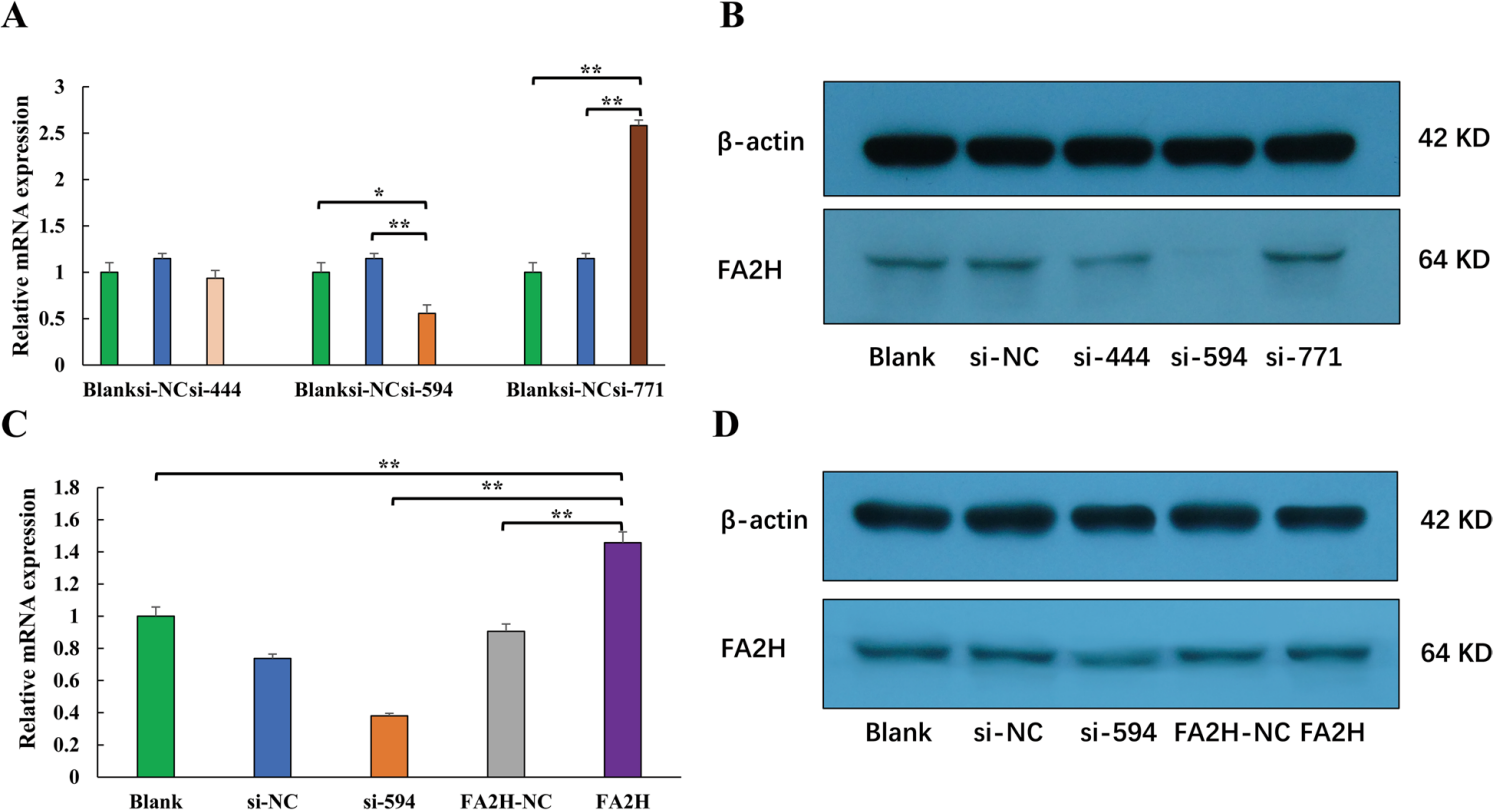
Detection of *FA2H* expression levels in DPCs after treatment. "Blank" represents the untreated control group; si-NC represents the *FA2H* gene interference control group. si-444, si-594, and si-771 represent the *FA2H* gene interference treatment groups, respectively; FA2H-NC represents the *FA2H* gene overexpression control group; pCDNA3.1( +)-FA2H represents the *FA2H* gene overexpression treatment group. Data are the mean ± SEM, and statistical analysis was performed using a T-test. * represents a significant difference, *p* < 0.05, ** represents a very significant difference, *p* < 0.01

The three synthesized *FA2H* gene interference fragments were transfected into DPCs, and the mRNA expression of *FA2H* was detected by qRT-PCR. The results showed that si-594 had the best interference effect, followed by si-444, and si-771 had no interference effect on the *FA2H* gene (Fig. 6A). The FA2H protein expression detected by Western blot was consistent with the mRNA results (Fig. 6B). Therefore, we selected si-594 as the *FA2H* gene interference fragment for subsequent experiments.

#### *FA2H* gene overexpression effect detection

The cDNA sequence of the goat *FA2H* gene was successfully cloned, and the full length was 1131 bp. We constructed the eukaryotic expression vector of the *FA2H* gene: pCDNA3.1( +)-FA2H. Through qRT-PCR detection, it was found that the overexpression effect of the *FA2H* gene was 145.80 ± 22.00%, and the inhibitory effect of the si-594 interference fragment was 38.10 ± 5.20% (Fig. 6C). The FA2H protein expression was found to be consistent with the mRNA expression trend by Western blot detection (Fig. 6D).

#### Effects of *FA2H* gene on DPCs

**Fig. 7:**
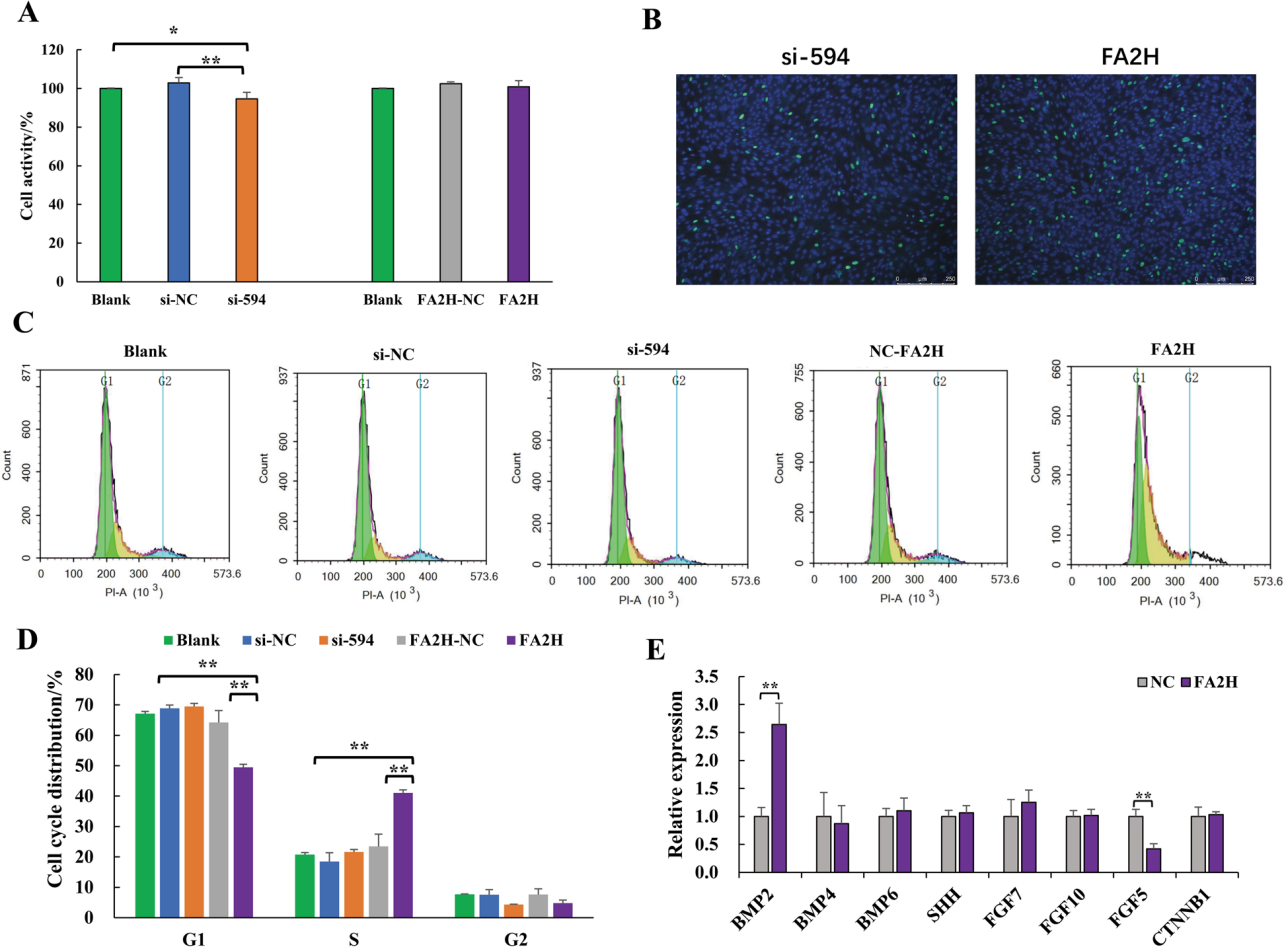
The effect of the *FA2H* gene on the DPCs of cashmere goats. **A**. Cell activity detection; **B**. Cell proliferation detection; **C**, **D**. Cell cycle detection; **E**. Gene expression after *FA2H* overexpression in DPC; Data are the mean ± SEM, and statistical analysis was performed using a T-test. * represents a significant difference, *p* < 0.05, ** represents a very significant difference, *p* < 0.01

The CCK-8 assay showed that the cell viability was 102.92 ± 2.73% in the si-NC group, 94.59 ± 3.42% in the si-594 group, 102.46 ± 1.11% in the FA2H-NC group, and 100.90 ± 3.15% in the overexpression group. Compared with the si-NC group, interfering with *FA2H* gene expression significantly decreased the activity of DPCs (*P* < 0.01). There was no significant difference in the activity of DPCs between the FA2H-NC group and the overexpression group (*P* > 0.05) (Fig. 7A).

Cell proliferation was detected by EDU. The results showed that the nuclei of all cells were stained blue, and the proliferating cells were stained green. It can be seen from Fig. 7B that overexpression of the *FA2H* gene can promote the proliferation of DPCs.

The results of the dermal papilla cell cycle detected by flow cytometry are shown in Fig. 7C and D. Disturbing the *FA2H* gene had no obvious effect on the dermal papilla cell cycle. Compared with the blank control group and the FA2H-NC group, the proportion of cells in the G1 phase was significantly decreased (*P* < 0.01), the proportion of cells in the S phase was significantly increased (*P* < 0.01), and the proportion of cells in the G2 phase did not change significantly when the *FA2H* gene was overexpressed. (*P* > 0.05). This shows that overexpression of *FA2H* can promote cells moving from the G1 phase to the S phase.

The expression levels of hair follicle-related genes were detected by qRT-PCR (Fig. 7E). Compared with the control group, the expression of the *FGF5* gene in the DPCs of the overexpression group was extremely significantly downregulated (*P* < 0.01), the expression of *BMP2* was extremely significantly upregulated (*P* < 0.01), while the expression levels of *BMP4*, *BMP6*, *SHH*, *FGF10,* and *CTNNB1* were not significantly changed (*P* > 0.05).

## Discussion

### Transcriptome sequencing data analysis

In this study, we collected skin tissues during the secondary hair follicle growth period of Jiangnan cashmere goats from coarse and fine cashmere groups. A total of 479 DEGs were identified in the Fe and Ce groups. In the differentially expressed gene analysis, we focused on genes annotated on hair follicles, hair-related GO terms and KEGG signaling pathways.

Mammalian skin and its appendages are derived from the mesoderm and ectoderm during embryogenesis. The embryonic surface appears as a single layer of epithelial cells and the dermis is made up of fibroblasts. The epidermal–dermal interaction induces hair follicle formation [15], so we have focused on some genes annotated to epidermal cell differentiation, development, and spreading (GO: 0,009,913, GO: 0,008,544, GO: 0,035,313), including *GLI1*, *KLF4* and *COL5A1*. Three DEGs, *CAV1*, *KLF4,* and *GRN*, are annotated to epithelial cell differentiation, proliferation and migration (GO: 0,030,857, GO: 0,010,632, GO: 0,050,678). *COL5A1* encodes an alpha chain for a low-abundance fibrillar collagen. The *COL5A1* gene plays a role in encoding type V collagen. Collagen is a family of proteins that strengthen and support many tissues in the body, including the skin, bones, and ligaments [16]. *CAV1* is able to translate caveolin-1, a protein that appears to have multiple functions in cells and tissues throughout the body. Studies have shown that vesicles are especially abundant in fat cells, the cells that store fat for energy. Fat cells make up most of the adipose tissue in the body. In these cells, vesicles appear to be critical for the normal transport, processing and storage of fat [17]. *KLF4* is a zinc-containing transcription factor that protects endothelial cells, regulates vasodilation, and is associated with inflammation and oxidative stress [18, 19]. *GRN* can encode granulin precursor. Granulin is considered to be related to many autoimmune diseases [20]. There was no clear evidence in the current study that *GLI1*, *KLF4*, *COL5A1*, *CAV1*, and *GRN* can affect cashmere traits.

Some cytokines also play an important role in hair follicle growth, such as vascular endothelial growth factor (VEGF), which is involved in regulating blood vessel formation. VEGF promotes the growth of blood vessels during the growth phase of the hair follicle to meet the high nutritional needs of the hair follicle [21]. We found four DEGs were annotated as functionally related to blood vessel endothelial cell migration (GO: 0,043,535, GO: 0,043,537, GO: 0,043,536, GO: 0,043,534 and GO: 0,043,536), including *KLF4*, *CD40*, *SOX18*, and *HSPB1*. Among them, early studies have shown that *SOX18* is involved in the regulation of hair follicle development [22, 23]. The study further confirmed our speculation that *SOX18* could affect the fiber diameter of cashmere goats.

In our results, some keratin family genes were found to be highly expressed in Jiangnan cashmere goat skin tissue, including *KRTAP 7–1*, *KAP8*, *KRTAP 13.1*, *KRTAP 6–1*, *KRT25*, *KRT27*, *KRT5*, *KRTAP 11–1*, *KRTAP 3–1,* and *KRT14*. It is noteworthy that eight keratin family genes were differentially expressed in the Ce and Fe groups, including *LOC102168573*, *LOC108636556*, *LOC102188618*, *KAP8*, *LOC100861174*, *LOC10218515*0, *KRT36,* and *KRT7*. *KAP8* is a member of the ultra-high sulfur KAPs family. Yang et al. found that the expression of *KRTAP8-1* was significantly increased in the secondary hair follicle growth phase of Inner Mongolia cashmere goats [24]. Another study found that the expression of the KRTAP8-2 gene in the secondary hair follicles of Liaoning cashmere goats was significantly higher than that in the primary hair follicles, according to qRT-PCR detection [25]. *KRTAP8-2* polymorphisms were found to be associated with wool length in five sheep breeds in Pakistan [26]. *KTR36* belongs to type I acidic keratin. Early research found, using PCR-SSCP and Sanger sequencing technology in 512 Altay sheep, that the mutation site of the *KRT36* gene was significantly correlated with wool fiber diameter, net wool rate, and length (*P* < 0.01) [27]. In addition, in a study of Chinese Merino sheep, it was found that there is a moderately polymorphic SNP locus in the exon of the *KRT36* gene, and this SNP can significantly affect wool fineness (*P* < 0.05) [28]. *KRT7* belongs to type II keratin, which is associated with various diseases in humans [29]. However, there are few studies on *KRT7* related to hair follicles and hair, and its specific mechanism of action on cashmere traits is still unclear.

Activation of signaling pathways such as WNT, MAPK, NOTCH, HEDGEHOG, and TGF-β in the skin is necessary for the normal development of hair follicles and hair phenotypes [30, 31]. The Wnt/β-catenin signaling pathway plays an important role in the induction of hair follicle formation and is extensively involved in all aspects of hair follicle morphogenesis and periodic growth, especially in the initial stage of hair follicle development [32]. In our study, the differentially expressed gene *WNT5A* was classified as an atypical Wnt family member [32, 33], which is associated with human skin diseases such as psoriasis [29], papulosquamous skin disease [34], and non-melanoma skin cancer [35]. Studies in mice have shown that *WNT5A* regulates the hair follicle differentiation and cycle through the mechanism of epithelial-mesenchymal interaction [33, 36]. *IGFBP4* is a member of the insulin-like growth factor binding protein family, which can participate in the WNT signaling pathway [37]. In our protein interaction network (Fig. 5B), *WNT5A* and *IGFBP4* were found to act on *FZD8*, but how they affect cashmere traits of cashmere goats has not been clearly explained.

We compared the DEGs obtained in this study with previous cashmere goat studies. Wang, F. H. et al. detected 7 gene polymorphicities related to cashmere yield in 1920 Inner Mongolia cashmere goats by 70 K chip detection, among which *AKR1B1* gene was highly expressed in skin tissues of Ce group of Xinjiang cashmere goats. These results suggest that *AKR1B1* gene may regulate cashmere fineness traits in cashmere goats [38]. In the study of Tibetan cashmere goats, 18 genes similar to our research results were identified by transcriptome sequencing to be differentially expressed in skin tissues of different cashmere finness, among which *SOX4* gene was the focus of our attention [5]. *SOX4* is an important developmental transcription factor that regulates multiple pathways including stem cell differentiation and progenitor cell development, such as through PI3K, WNT, and TGFβ signaling. *SOX4* interacts with a variety of other transcription factors, thereby influencing the expression environment and tissue specificity of genes [39]. Foronda et al. used gene knockout technology and found that the *SOX4* gene of hair follicle stem cells in mice was completely inactivated, showing that the skin repair mechanism was disabled and the skin was prematurely aged [40]. Kobielak K et al. found that the *SOX4* gene was involved in the formation of hair shafts in mice [41]. Another study in mice found that deletion of the *SOX4* gene delayed hair growth [42]. This series of studies showed the importance of the *SOX4* gene in hair follicles and hair. In addition, transcriptome sequencing found that some differential genes, including *FA2H*, *THBS2* and *KAP8*, were related to cashmere fineness in Liaoning cashmere goats[2, 4]. It is noteworthy that these three genes were all differentially expressed in our study.

### Regulation of the *FA2H* gene in DPCs

It is worth noting that *FA2H* was annotated on GO entries such as sebaceous gland cell differentiation (GO: 0,001,949), cell proliferation regulation (GO: 0,042,127), hair cycle regulation (GO: 0,042,634) and skin barrier establishment (GO: 0,061,436). Referring to previous studies, we used qRT-PCR to verify the high expression of the *FA2H* gene in the degenerative stage of secondary hair follicles of Jiangnan cashmere goats. This result is consistent with the findings of study of Shanbei white cashmere goats [43].

In order to explore the role of the *FA2H* gene in hair follicles, we found that the overexpression of the *FA2H* gene could promote the proliferation of DPCs by transient transfection. The DPCs are the "signaling centers" of the hair follicle [14]. Two components of growing hair follicles contain blood vessels: dermal papillae and connective tissue sheaths. The papillary layer of the dermis is continuous with the connective tissue sheath to form the outermost cell layer surrounding the hair follicle [44, 45]. Active DPCs encourage blood vessels to supply nutrients to hair follicles. Abundant nutrition also is the hair follicle grows hair better fundamental guarantee [46, 47]. In our transcriptome data, *FA2H* gene was up-regulated in Ce group. This further proves that the expression of *FA2H* gene is positively correlated with cashmere fiber diameter. However, how the *FA2H* gene plays a regulatory role in DPCs remains unclear. Some signaling pathways, including FGF, WNT, SHH, and BMPs, are the main signaling pathways in the development of hair follicles, and they work together in coordination with each other [31, 48, 49]. Therefore, we explored the effect of the *FA2H* gene on the expression of major genes in these four signaling pathways on DPC. Studies have shown that overexpression of the *FA2H* gene can up-regulate the expression of the *BMP2* gene and down-regulate the expression of *FGF5* gene. The BMPs signaling pathway is involved in maintaining DPC activity and hair follicle regeneration. *FGF5* expression is elevated at the end of the anagen phase, and *FGF5* deletion prolongs the secondary anagen phase, resulting in longer hair. These results suggest that *FA2H* gene can promote dermal papilla cell activity and hair growth by regulating the expression of *BMP2* and *FGF5* genes.

## Conclusion

Transcriptome sequencing was performed on the skin tissues of eight Jiangnan cashmere goats based on cashmere fineness. There were 479 DEGs in Ce group and Fe group. Seven DEGs (*SOX18*, *SOX4*, *WNT5A*, *IGFBP4*, *KAP8*, *KRT36,* and *FA2H*) were speculated to be related to cashmere fineness. Through qRT-PCR, Western blot, CCK-8, EDU, and flow cytometry, it was found that *FA2H* could promote the proliferation of DPCs in cashmere secondary hair follicles, and affect cashmere traits by regulating the expression levels of *FGF5* and *BMP2*.

## Methods

### Experimental animals

The experimental site was the Xinjiang Aksu White Tiger Cashmere Goat Breeding Center. The experimental animals were eight adult female Jiangnan cashmere goats with the same body weight and feeding conditions. Eight experimental goats had the same father (half sibling, genetic relationship = 0.25). Cashmere samples were cut from the left scapula 10 cm from the back edge of the experimental animals. Then the cashmere samples were sent to the Xinjiang Autonomous Region Wool and Cashmere Quality and Safety Supervision and Inspection Center, and the related indicators of the cashmere fiber diameter were determined by the fiber diameter optical analyzer OFDA 2000. According to the mean fiber diameter (MFD) of the cashmere, the experimental animals were divided into two groups, the fine cashmere group (Fe, *n* = 4) and the coarse cashmere group (Ce, *n* = 4). The skin tissue from the eight experimental animals was collected during the anagen phase (September) of secondary hair follicles for transcriptome sequencing. The skin tissues of three of the experimental animals (Fe1, Fe2, and Fe3) were collected in the anagen phase (September), the regression phase (January), and the growth phase (March) of the secondary hair follicle to verify the *FA2H* gene. Skin samples were collected from shoulder blades of cashmere goats with a 10 mm diameter skin sampler. The skin tissues were rinsed with PBS and immediately stored in liquid nitrogen.

### RNA Extraction and Sequencing

Total RNA was extracted from eight cashmere goat skin tissue samples using TRIzol. The extracted total RNA was tested for purity, concentration and integrity using an Agilent Bioanalyzer 2100 and NanoDrop One spectrophotometer. The 1 ug total RNA was taken from each sample to construct the mRNA library. The constructed library was quality checked by the Qsep-400 method. Paired-end sequencing of eight mRNA libraries was performed on an Illumina NovaSeq 6000 sequencer.

### Quality Control and annotation

In order to obtain clean reads that could be used for data analysis, the raw reads were filtered using FastQC software. According to the goat reference genome and annotation files in the Ensembl database (download at: ftp://ftp.ensembl.org/pub/release93/fasta/capra_hircus/dna/Capra_hircus.ARS1.dna.toplevel.fa.gz), using HISAT2 [50], we mapped the clean reads of the Jiangnan cashmere goat to the goat reference sequence (Capra hircus ARS 1.97). Based on the mapping results, the genome was assembled using StringTie, and both known and novel genes were identified [51]. Known genes whose sequences can be annotated to the reference genome. Novel genes are defined as sequences not annotated to the reference genome, and these sequences do not contain less than 50 amino acid residues or contain only a single exon.

### Functional analysis of DEGs

FPKM was used as an indicator to measure the level of transcript or gene expression [52]. The sequenced sequences were aligned to the genome and the sample data were normalized using the software StringTie package [51]. DESeq2_EBSeq package was used to calculate the DEGs between Fe and Ce groups [53]. Differentially expressed mRNAs were analyzed by GO and KEGG [54–56]. Candidate genes encoding functional protein–protein interactions (PPIs) were investigated through the STRING Genomics 11.0 database [57], and PPI networks with scores greater than 0.7 were retained. Finally, the PPI network was visualized using Cytoscape 3.6.1 software [58].

### *FA2H* expression verification

In order to identify the reliability of the sequencing data, the *FA2H* gene and five random DEGs were selected, and primers were designed using Primer 5.0 with reference to the CDS sequence published on NCBI (Table S3). Using *GAPGH* as a housekeeping gene, qRT-PCR was used to verify the expression levels of the eight genes in the rough and thin skin tissues of Jiangnna cashmere goats. The total RNA of eight cashmere goat skin tissue samples was reverse transcribed according to the instructions of StarScript II First-Strand cDNA Synthesis Kit-II (GenStar, A222-02). According to the instructions of 2 × RealStar Green Fast Mixture (GenStar, A301-05), real-time quantitative PCR was performed on eight cDNAs in the Bio-Rad CFX96 Real-Time PCR system. Similarly, qRT-PCR was used to verify the expression level of the FA2H gene in the skin tissue of the three stages of secondary hair follicles.

In order to detect the expression of the FA2H gene in DPCs, the DPCs isolated from the secondary hair follicles of Jiangnan cashmere goats were cultured. Fourth-generation DPCs were used in all subsequent validation tests. The dermal papilla cell culture medium was DMEM/F12 containing 10% fetal bovine serum. The culture environment was 37℃ Celsius in an incubator containing 5% carbon dioxide. The fourth-generation DPCs in good condition were seeded into 6-well culture dishes at 1*10^5^ cells per well. When the cell confluence reached about 70%, the culture medium was discarded, and the cells were washed three times with PBS. Then, the expression of FA2H in the DPCs was detected according to the instructions of the SABC-CY3 immunohistochemical staining kit (Boster Biological Technology Co. Ltd, SA1074). The following antibodies were used: 1:100 FAAH1 Polyclonal Antibody (Bioss, bs-5104R).

### Screening of *FA2H* gene interference fragments

Small interfering RNA (siRNA) is a class of double-stranded RNA molecules that are 20–25 base pairs in length. SiRNA can block the transcription or translation of a specific gene to inhibit gene expression [59, 60]. Therefore, three sets of siRNAs were designed with reference to the CDS sequence of the goat *FA2H* gene (XM_018061705.1) and synthesized by Shanghai Bioengineering Company. The siRNA sequences are shown in Table S4. The DPCs of cashmere goat were seeded into 6-well culture dishes at 1*10^5^ cells per well. When the cells reached about 70% confluence, siRNA was transfected into DPCs using Lipofectamine™ 3000 reagent. The experimental groups were as follows: blank control, transfected si-NC, and transfected siRNA. We repeated three wells for each grouping. The culture medium was replaced with fresh medium 6 h after transfection. After 48 h, the total cell RNA was extracted with TRIzol. The mRNA expression of *FA2H* was detected by qRT-PCR. After 72 h, the total cell protein was extracted with RIPA lysis solution (Biyuntian, P0013B), and the BCA protein concentration assay kit (Biyuntian, P0010S) was used to detect the protein concentration. The protein expression of FA2H was detected by Western blot. The following antibodies were used: 1:1000 Rabbit Anti-beta-Actin Polyclonal Antibody (Bioss, bs-0061R) (Immunoway, YM3028); 1:1000 FAAH1 Polyclonal Antibody (Bioss, bs-5104R); 1:1000 Anti-rabbit IgG H&L/HRP (Bioss, bs-0295 M-HRP).

### Construction of the overexpression vector

Overexpression of FA2H gene by recombinant plasmid can help to explain the regulatory role of target gene in DPCs. Cloning was performed with reference to the CDS sequence of the goat *FA2H* gene (XM_018061705.1). Clone primer information is shown in Table S5. The target fragment and pcDNA3.1( +) vector were digested with NheI and XhoI enzymes in a water bath at 37℃ for 4 h. The digested product was detected by agarose gel electrophoresis, and the target band was recovered by a gel recovery kit. The target fragment was ligated with the vector overnight at 4℃. The overexpression vector was obtained after the ligation: pCDNA3.1( +)-FA2H.

To test the overexpression effect of pCDNA3.1( +)-FA2H, siRNA and plasmid were transfected into DPCs by Lipofectamine™ 3000 reagent. The experimental groups were as follows: blank control, si-NC, si-594, FA2H-NC, pCDNA3.1( +)-FA2H, and each group was repeated three times. After 48 h, the cells were collected to detect the mRNA expression level of *FA2H* by qRT-PCR technology, and 72 h later, the cells were collected to detect the protein expression of FA2H by Western blot.

### Cell proliferation assay

The effect of *FA2H* gene on DPC activity was detected by CCK-8 kit. DPCs were seeded into 96-well plates at 5 × 10^3^ cells per well. The experimental groups were as follows: blank control, si-NC, si-594, FA2H-NC, pCDNA3.1( +)-FA2H, and five replicates were set for each group. After 12 h, 10 ul of CCK8 reagent was added to each well to react for 4 h, and the OD value was detected by a microplate reader at 450 nm.

DPCs were seeded into 24-well plates at 1 × 10^4^ cells per well, and si-594 and pCDNA3.1( +)-FA2H were transfected with three replicates per well. After 24 h, the proliferation efficiency was detected according to the operation steps of the Edu-488 cell proliferation detection kit (Biyuntian, C0071S).

DPCs were seeded into 6-well plates at 1 × 10^5^ cells per well. The experimental groups were as follows: blank control, si-NC, si-594, FA2H-NC, and pCDNA3.1( +)-FA2H, and each group was repeated three times. After 24 h, the cells were processed according to the instructions of the cell cycle kit (Keygen, KGA511-KGA512), and the red fluorescence at the excitation wavelength of 488 nm was recorded by flow cytometry. Data were analyzed with FlowJo software (V10.6.2).

### Expression level detection of hair follicle genes

We designed primers for the CDS regions of genes related to folliculogenesis, development, and cycle, including *BMP2*, *BMP4*, *BMP6*, *SHH*, *FGF5*, *FGF7*, *FGF10* and *CTNNB1*. The qRT-PCR was used to detect the expression changes of these eight genes in DPCs after overexpression of the *FA2H* gene. The gene primer sequences are shown in Table S1.

### Statistical analysis

The non-parametric test in SPSS 19.0 software was used to analyze the significant differences in body weight and fiber diameter-related traits of experimental goats in different groups. When *P*-value < 0.05 and the Fold change > 1, the differentially expressed genes between Ce group and Fe group will be identified. The 2 − ΔΔCt method was used to analyze the relative mRNA expression levels [61].

## Supplementary Information

**Additional file 1: Table S1**. RNA-Seq quality control result. **Table S2**. Reference genome alignment read statistics. **Table S3**. Primer information. **Table S4**. Sequence information of FA2H gene interference fragments. **Fig. S1** Comparison of FPKM density distribution of each sample. Different colors in the figure represent different samples, the abscissa represents the logarithm of the FPKM of the corresponding sample, and the ordinate represents the probability density. **Fig. S2** FPKM boxplot of each sample. The abscissa in the figure represents different samples; the ordinate represents the logarithm of the FPKM expression of the samples. **Fig. S3** Untrimmed original image of Figure 7B. A. Anti-beta-Actin Polyclonal Antibody; B. FAAH1 Polyclonal Antibody. Different lanes 1-5 are represented as Blank, si-NC, si-444, si-594, and si-771 respectively. **Fig. S4** Untrimmed original image of Figure 7D. A. Anti-beta-Actin Polyclonal Antibody; B. FAAH1 Polyclonal Antibody. Different lanes 1-5 are Blank, si-NC, si-594, FA2H-NC, and FA2H respectively.

## Data Availability

All raw data from transcriptome sequencing are publicly available in the SRA database (Accession numbers: PRJNA778726). The original data download address to https://www.ncbi.nlm.nih.gov/sra?term=PRJNA778726&cmd=DetailsSearch.
