# Supplementary material for: Effect of the FA2H Gene on cashmere fineness of Jiangnan cashmere goats based on transcriptome sequencing
Source: BMC Genomics. 2022 Jul 21;23:527. doi: 10.1186/s12864-022-08763-7 (PMC9306159; doi:10.1186/s12864-022-08763-7)
Supplement: Supplementary file 1 — Additional file 1: Table S1. RNA-Seq quality control result. Table S2. Reference genome alignment read statistics. Table S3. Primer information. Table S4. Sequence information of FA2H gene interference fragments. Fig. S1 Comparison of FPKM density distribution of each sample. Different colors in the figure represent different samples, the abscissa represents the logarithm of the FPKM of the corresponding sample, and the ordinate represents the probability density. Fig. S2 FPKM boxplot of each sample. The abscissa in the figure represents different samples; the ordinate represents the logarithm of the FPKM expression of the samples. Fig. S3 Untrimmed original image of Figure 7B. A. Anti-beta-Actin Polyclonal Antibody; B. FAAH1 Polyclonal Antibody. Different lanes 1-5 are represented as Blank, si-NC, si-444, si-594, and si-771 respectively. Fig. S4 Untrimmed original image of Figure 7D. A. Anti-beta-Actin Polyclonal Antibody; B. FAAH1 Polyclonal Antibody. Different lanes 1-5 are Blank, si-NC, si-594, FA2H-NC, and FA2H respectively. [file 12864_2022_8763_MOESM1_ESM.pdf]

Table S1. RNA-Seq quality control result

Sample Name	Read Sum	Base Sum	GC/%	Q20/%	Q30/%
Ce-1	36,417,155	10,878,015,024	48.07	98.47	95.37
Ce-2	37,219,205	11,098,838,522	47.49	98.38	95.15
Ce-3	42,190,124	12,587,086,652	46.92	98.39	95.14
Ce-4	37,380,818	11,139,683,870	47.9	98.58	95.65
Fe-1	37,058,161	11,051,625,508	47.41	98.54	95.58
Fe-2	39,272,819	11,719,721,120	46.92	98.35	95.03
Fe-3	42,348,243	12,644,980,448	47	98.42	95.21
Fe-4	38,721,645	11,555,012,892	47.02	98.4	95.14

Note: Read Sum: total number of pair-end Reads in Clean Data; Base Sum: Clean Data Total Base number; GC: GC content of Clean Data, that is, the percentage of G and C bases in total bases in Clean Data; N: percentage of unrecognized bases in total bases in Clean Data; Q30: Clean Data Percentage of bases with a mass value greater than or equal to Q30.

Table S2. Reference genome alignment read statistics

Sample Name	Total Reads	Mapped Resds	Unique Mapped Reads	Multiple Mapped Reads	Reads Map to '+'	Reads Map to '-'
Ce-1	72,834,310	70,606,897 (96.94%)	68,742,561 (94.38%)	1,864,336 (2.56%)	34,844,100 (47.84%)	35,052,520 (48.13%)
Ce-2	74,438,410	71,999,939 (96.72%)	70,220,158 (94.33%)	1,779,781 (2.39%)	35,474,192 (47.66%)	35,714,193 (47.98%)
Ce-3	84,380,248	81,917,582 (97.08%)	80,047,193 (94.86%)	1,870,389 (2.22%)	40,455,337 (47.94%)	40,692,115 (48.22%)
Ce-4	74,761,636	72,677,614 (97.21%)	70,419,223 (94.19%)	2,258,391 (3.02%)	35,551,298 (47.55%)	35,962,380 (48.10%)
Fe-1	74,116,322	71,933,700 (97.06%)	70,038,566 (94.50%)	1,895,134 (2.56%)	35,414,125 (47.78%)	35,686,086 (48.15%)
Fe-2	78,545,638	75,901,736 (96.63%)	74,231,526 (94.51%)	1,670,210 (2.13%)	37,522,695 (47.77%)	37,727,620 (48.03%)
Fe-3	84,696,486	82,034,935 (96.86%)	80,090,317 (94.56%)	1,944,618 (2.30%)	40,493,218 (47.81%)	40,734,236 (48.09%)
Fe-4	77,443,290	75,262,378 (97.18%)	73,391,963 (94.77%)	1,870,415 (2.42%)	37,116,153 (47.93%)	37,340,760 (48.22%)

Note: ID: uniform serial number of samples; Total Reads: number of Clean Reads, by single end; The number of Reads Mapped into the reference genome and the percentage of Clean Reads. Uniq Mapped Reads: Number of Reads Mapped to unique locations in the reference genome and percentage of Clean Reads The number of Multiple Mapped Reads in the reference genome and the percentage in Clean Reads. Reads Map to '+' : the number and percentage of Clean Reads compared to the reference genome plus strand. Reads Map to '-' : compares the number of negative links of Reads to the reference genome and the percentage of Clean Reads.

Table S3. Primer information

Gene Name	Sequence (5’-3’)	Product Length/bp	ID
LOC108635406	F: TCCTTCTCTTTGCGGCGGAAG<br>R: AATGAGATTGTGGAAGATGCCCG	167	XM_018045561.1
MUCL1	F: GTGGCAGTTTCCACCTTCCT<br>R: ATCAACAGTGGTTGTATCAGAGG	90	XM_018049088.1
LOC108636093	F: ATGCCTCACTTCTATATTGCCC<br>R: CCACAGTGGTTGTATCAGAGGC	90	XM_018049017.1
LOC106503216	F: GGTGTGTCCAGCTGCTACC<br>R: CACAGACTGGCAACACCG	171	XM_018065078.1
LOC102173780	F: GAGCTGCAGAGGTCCTGTTT<br>R: GTGGGTCACAGCTGGATGAT	101	XM_018065045.1
FA2H	F: CGAGCACTCAGAAGACAG<br>R GCCATAGTAGGACCAACTG	266	XM_018061705.1
BMP2	F: ACAACCCGGCGATTCTTCTT<br>R: TTGGAGTTAGCTGTGGCAGG	173	NM_001287564.1
BMP4	F: CTCAACCAACCACGCCATTG<br>R: CGGCAACCACATCCCTCTAC	174	NM_001285646.1
BMP6	F: CTTCTTCAAGGCCAGTGAGGT<br>R: TGCTGTTGTAGTCTGCTGAGG	137	XM_018038922.1
SHH	F: GGAGTCGCTGCACTATGAGG<br>R: TCACTGAGCAGTGGATGTGC	149	XM_018046646.1
FGF7	F: TGACATGGATCCTGCCAAGTT<br>R: AGCCATTTGCTCTGGAGTCAT	107	XM_005685797.3
FGF10	F: GCATGTGCGGAGCTACAATC<br>R: TACCGCTGACCTTTCCGTTC	104	XM_005694730.3
FGF5	F: TGCTGTGTCTCAGGGGATTG<br>R: TGTGTATCGCGGAGGCATAG	167	NM_001291973.1
β-Catenin	F: ACACAGTTCGATGCTGCTCA<br>R: GATTGCACGTGTGGCAAGTT	126	XM_018066894.1
GAPDH	F:GGCGTGAACCACGAGAAGTAT<br>R:ATGGCGTGGACAGTGGTCAT	143	XM_005680968.3

Table S4. Sequence information of *FA2H* gene interference fragments

Sequence Name	Sequence (5'-3')
FA2H-si-444	F: GCCACUUGGGAGAGAAGUATT
	R: UACUUCUCUCCCAAGUGGCTT
FA2H-si-594	F: GGUCCUACUAUGGCGCCUUTT
	R: AAGGCGCCAUAGUAGGACCTT
FA2H-si-771	F: GCGACAGCGCUUACCUCAUTT
	R: AUGAGGUAAGCGCUGUCGCTT
si-NC	F: UUCUCCGAACGUGUCACGUTT
	R: ACGUGACACGUUCGGAGAATT

Table S5. Primers information for *FA2H* gene cloning

Gene name	Sequence (5'-3')
FA2H	F: ACTCACTATAGGGAGACCCAAGCTGGCTAGCATGGCC
	R: AGCGGGTTTAAACGGGCCCTCTAGACTCGAGTCACTGCGTCTTCGG

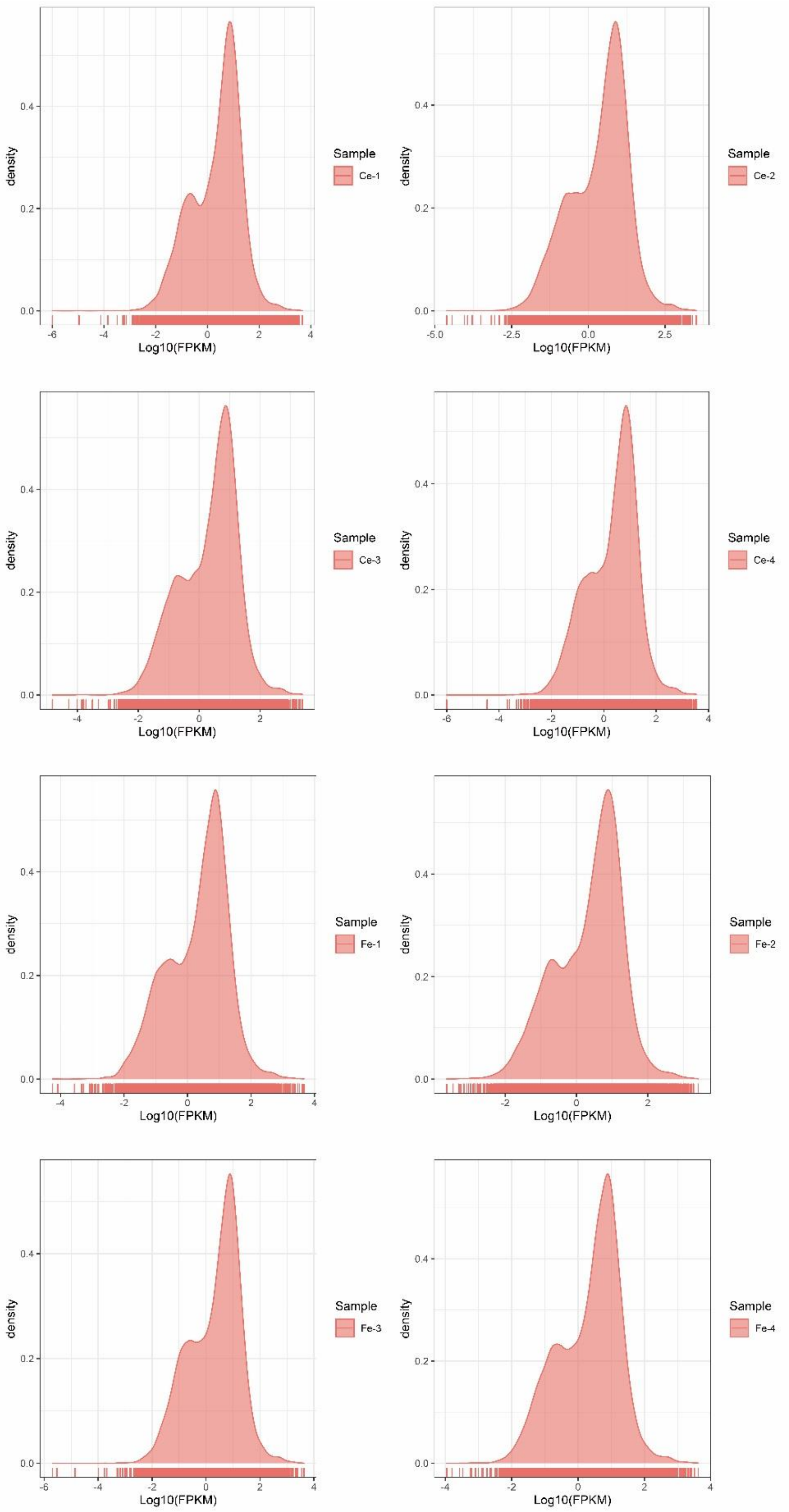

Fig. S1 Comparison of FPKM density distribution of each sample. Different colors in the figure represent different samples, the abscissa represents the logarithm of the FPKM of the corresponding sample, and the ordinate represents the probability density.

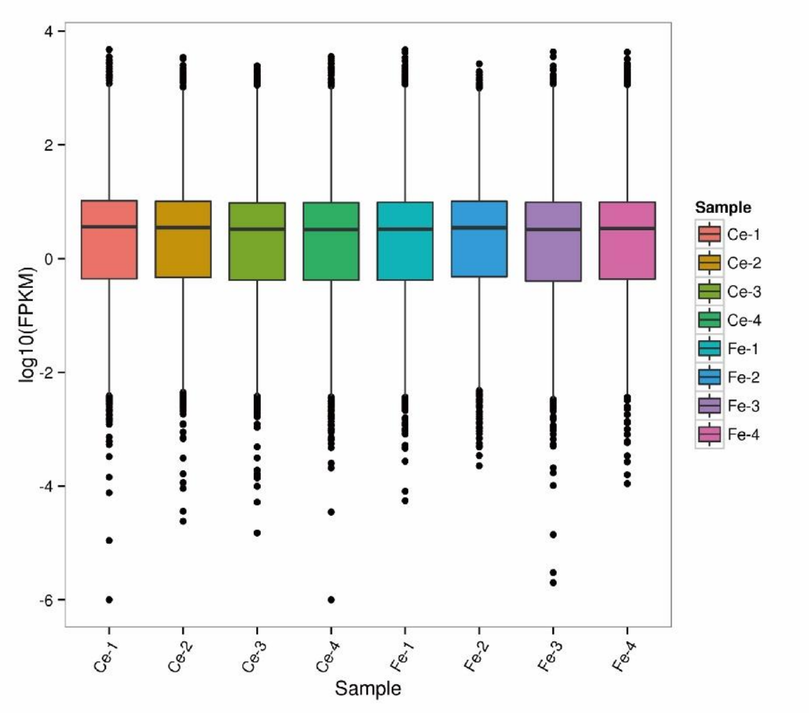

Fig. S2 FPKM boxplot of each sample. The abscissa in the figure represents different samples; the ordinate represents the logarithm of the FPKM expression of the samples.

A B

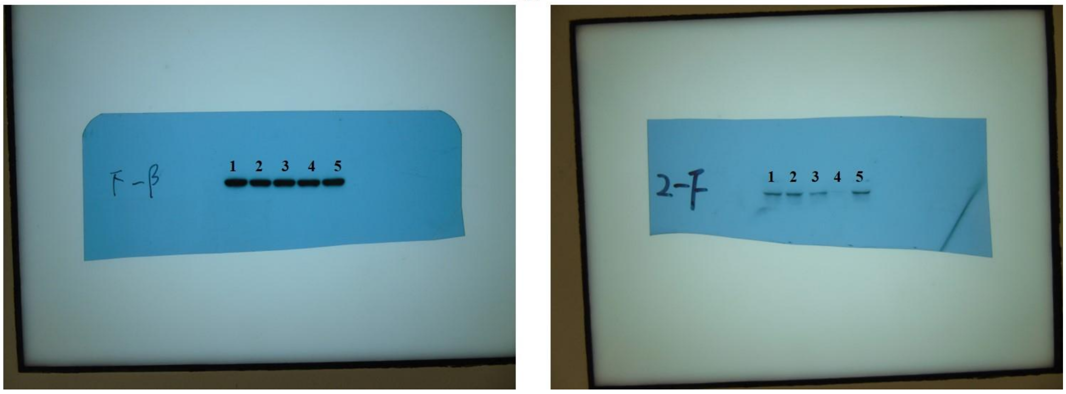

Fig. S3 Untrimmed original image of Figure 7B. A. Anti-beta-Actin Polyclonal Antibody; B. FAAH1 Polyclonal Antibody. Different lanes 1-5 are represented as Blank, si-NC, si-444, si-594, and si-771 respectively.

A B

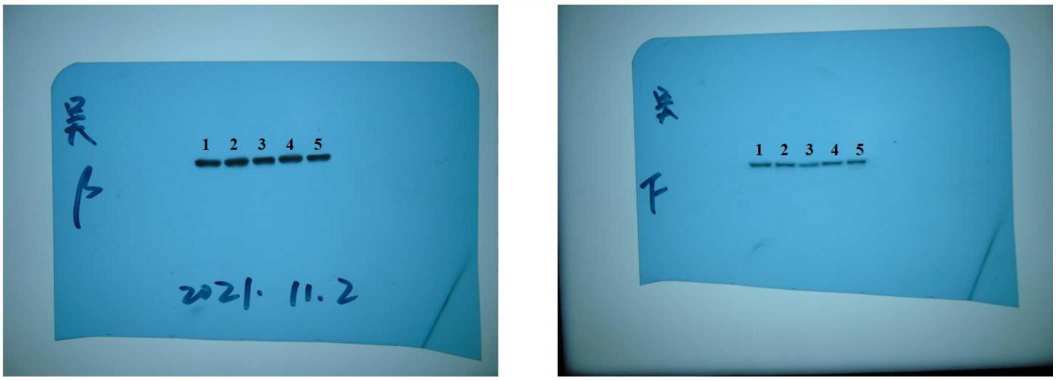

Fig. S4 Untrimmed original image of Figure 7D. A. Anti-beta-Actin Polyclonal Antibody; B. FAAH1 Polyclonal Antibody. Different lanes 1-5 are Blank, si-NC, si-594, FA2H-NC, and FA2H respectively
